# ChromoZoom: a flexible, fluid, web-based genome browser

**DOI:** 10.1093/bioinformatics/bts695

**Published:** 2012-12-06

**Authors:** Theodore R. Pak, Frederick P. Roth

**Affiliations:** ^1^Donnelly Centre, University of Toronto, Toronto, ON M5S3E1, ^2^Samuel Lunenfeld Research Institute, Mt. Sinai Hospital, Toronto, ON M5G1X5, ^3^Department of Molecular Genetics, ^4^Department of Computer Science, University of Toronto, Toronto, ON M5S3E1, Canada and ^5^Center for Cancer Systems Biology, Dana-Farber Cancer Institute, Boston, MA 02115, USA

## Abstract

**Summary:** Current web-based genome browsers require repetitious user input to scroll over long distances, alter the drawing density of elements or zoom through multiple orders of magnitude. Generally, either the server or the client is responsible for the majority of data processing, resulting in either servers having to receive and handle data relevant only to one user, or clients redundantly processing widely viewed data. ChromoZoom pre-renders and caches general-use tracks into tiled images on the server and serves them in an interactive web interface with inertial scrolling and precise, fluent zooming via the mouse wheel or trackpad. Custom tracks in several formats can be rendered by client-side code alongside the pre-rendered tracks, minimizing server load because of user-specific rendering and eliminating the need to transmit private data. ChromoZoom thereby enables rapid and simultaneous exploration of curated, experimental and personal genomic datasets.

**Availability:** Human and yeast genome researchers may browse recent assemblies within ChromoZoom at http://chromozoom.org/. Source code is available at http://github.com/rothlab/chromozoom/.

**Contact:** fritz.roth@utoronto.ca

**Supplementary information:** Supplementary data are available at *Bioinformatics* online.

## 1 INTRODUCTION

Genome browsers have become an essential tool for experimental and computational biologists. Among well-known web-based browsers, the UCSC Genome Browser has gained popularity for its ready availability, comprehensive library of genomes and curated data and the ability to display custom data uploaded by researchers (Dreszer *et al.,* 2011; Nielsen *et al.*, 2010). Overlaying experimental data (e.g. sequence variation) onto curated tracks (e.g. gene predictions) allows for the formulation and verification of biological hypotheses. Researchers unfamiliar with a newly encountered locus can inspect it within a genome browser to determine the gene layout or potential regulatory elements and design polymerase chain reaction or other experiments.

Although many researchers have become familiar with the UCSC browser and others like GBrowse (Stein *et al.*, 2002), Ensembl (Stalker *et al.*, 2004) and the NCBI Map Viewer (Wheeler *et al.*, 2003), these tools originated before the advent of modern web interfaces that use less page transitions and more data dynamically loaded using Asynchronous JavaScript and extensible mark-up language (AJAX) and HTML5 techniques. Some have attempted to incorporate aspects of the ‘rich internet application’ experience into their interfaces—for example, each of the aforementioned browsers now allow the user to drag the track to move the current view—but none had a user interface allowing smooth navigation equivalent to Google Maps, for example, animated transitions while zooming and the absence of loading interruptions throughout all navigational operations.

In response, ‘next-generation’ browsers like AnnoJ (http://www.annoj.org), JBrowse (Skinner *et al.,* 2009) and ABrowse (Kong *et al.*, 2012) have been built to take advantage of modern web technologies, adding more seamless interactions that preserve the user’s sense of location while traversing the massive ‘landscape’ of a genome. AnnoJ and JBrowse render most genomic data on the browser-side, the former drawing pixels on HTML5 Canvas elements and the latter manipulating standard HTML elements. However, both require preprocessing of custom data by an administrator before it can be rendered within the browser; neither accept flat files via the web interface in the manner of the UCSC browser. ABrowse renders all data to tiled images, but cannot zoom smoothly and requires custom data to be fully uploaded to the server—which can be prohibitive for large datasets. Finally, none of the browsers features inertial scrolling, a feature popularized by iOS and Google Maps whereby a scrollable surface can be ‘thrown’ with a finger or the cursor to move across long distances.

## 2 FEATURES

**Fig. 1.**
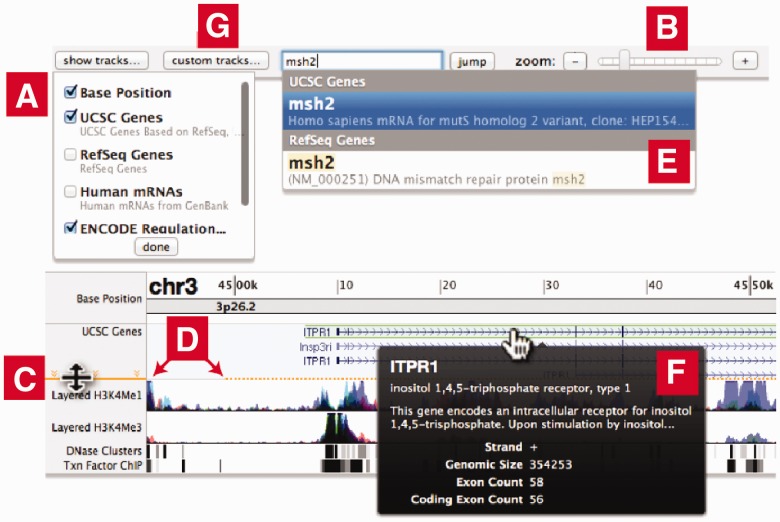
The ChromoZoom web interface. (**A**) Select tracks to be displayed. (**B**) Zoom from genome view to individual base pairs. (**C**) Resize tracks to automatically unpack features. (**D**) Orange line warns of cropped data. (**E**) Autocomplete for keyword searching. (**F**) Custom tooltips with feature details. (**G**) Add custom data tracks from local files or remote URLs

ChromoZoom attempts to improve the interactivity introduced by ‘next-generation’ genome browsers while adding custom data capabilities and the familiar rendering styles of established browsers like UCSC. The full-window interface can maximize use of vertical display space by wrapping tracks into multiple lines, much like paragraph text. General-use tracks can be added via a dropdown menu (Fig. 1A); the layout updates dynamically. The user can drag lines horizontally and vertically, and they can also ‘throw’ the line with the mouse to scroll smoothly through the genome. Motion of the tracks is *never* interrupted, preserving the user’s sense of location throughout all navigation operations.

To zoom in and out, the user can use a familiar slider and buttons (Fig. 1B) or keyboard shortcuts to move all the way from the genome-wide view to the base pair level, or they can position their mouse cursor and use a scroll wheel or trackpad to zoom continuously and precisely at any visible location (a feature unique to ChromoZoom). To see more detail, the user can vertically resize the track using its side label (Fig. 1C), which will ‘unpack’ individual elements and labels as completely as vertical space allows. An orange warning line appears if elements are being cropped by the edge of the track (Fig. 1D). Users can also reorder tracks by dragging the labels in the sidebar and remove them using the ‘show tracks…’ menu. A search bar allows users to specify coordinates or coordinate ranges, for example, ‘chr1:12340-12350’, or keywords like ‘MSH2’, which display a dropdown of matching features (Fig. 1E). Tooltips appear when the user hovers over track features (Fig. 1F), and a click loads a feature description page from the UCSC Browser. A full comparison of features with other current web-based genome browsers is provided in Supplementary Table S1.

ChromoZoom is the first online genome browser to provide client-side parsing *and* rendering of user-provided custom data, initiated by clicking the ‘custom tracks … ’ button (Fig. 1G). Browser Extensible Data (BED) tracks containing range-based features and ‘wiggle’ (WIG) tracks of continuous quantitative data, formatted according to UCSC’s guidelines (http://genome.ucsc.edu/FAQ/FAQformat.html), can be read from the client’s local disk or a public URL and are plotted adjacent to the normal tracks. The full suite of zooming, panning, reordering and expansion interactions applies equally to custom tracks. For large datasets, the user can provide a track line with a bigDataUrl pointing to a pre-indexed BED or WIG data file in bigBed/bigWig format (Kent *et al.*, 2010) or sequence variations in tabix-compressed Variant Call Format, VCFTabix, (Danacek *et al.*, 2011; Li, 2011), again formatted to UCSC’s guidelines, and the application will seamlessly fetch data for the current view with AJAX and render graphical data within the browser. ChromoZoom is, therefore, ideal for exploration of experimental data by researchers, enabling the visualization of custom results alongside a dynamic representation of curated genomic information.

## 3 IMPLEMENTATION

ChromoZoom uses a local installation of the UCSC Genome Browser to generate and pre-cache tiled PNG images (via Ruby scripts). Rake (‘Ruby make’) directs the creation of a configuration (YAML) file for each genome, the capture of tile images and the creation of a JSON file to initialize the web interface. A Ruby extension written in C maximizes image-processing performance. ImageMagick is used for many image operations and the Nokogiri library for HTML parsing. The eight tracks available on http://chromozoom.org/for the hg19 assembly and the 19 tracks for the sacCer3 assembly consume 64 GB and 5.4 GB of disk space, respectively. Tile generation scripts are run in parallel across a computing cluster.

Because all general-use track data are converted to static tile images, they can be served efficiently via an Apache web server, a small amount of PHP, and appropriate use of HTTP cache directives. Tokyo Tyrant, a fast on-disk hash table, stores the tile images and caches search queries. The front-end is constructed on top of jQuery and the jQuery UI widget framework. Many HTML5 features are used, such as SVG, the Canvas and File APIs and Web Workers. Web Workers allow computation on custom track data to be moved to independent JavaScript threads to avoid locking the browser UI. JavaScript for handling custom tracks has been designed for straightforward addition of new data formats and drawing styles.

The visual style is influenced by Edward Tufte’s principle of maximizing the data-ink ratio (Tufte, 2001). Repetitive high-order digits are removed from the Base Position track, the data are placed front and centre with minimalistic borders and labelling, and control widgets are kept in the margin and in collapsed format until activated by the user.

## Supplementary Material

Supplementary Data
