# Supplementary material for: ChromoZoom: a flexible, fluid, web-based genome browser
Source: Bioinformatics. 2012 Dec 6;29(3):384–6. doi: 10.1093/bioinformatics/bts695 (PMC3562068; doi:10.1093/bioinformatics/bts695)
Supplement: Supplementary Data [file supp_29_3_384__index.html]

ChromoZoom: a flexible, fluid, web-based genome browser — Supplementary Data 

# ChromoZoom: a flexible, fluid, web-based genome browser

## Supplementary Data

files

**Files in this Data Supplement:**

- Supplementary Data - doc file
