# Supplementary material for: ChromoZoom: a flexible, fluid, web-based genome browser
Source: Bioinformatics. 2012 Dec 6;29(3):384–6. doi: 10.1093/bioinformatics/bts695 (PMC3562068; doi:10.1093/bioinformatics/bts695)
Supplement: Supplementary Data [file supp_bts695_Pak_ChromoZoom_S1.doc]

**Table S1. Feature comparison with current published online genome browsers.**

|  | Panning | Zooming | Change track order | Add/remove tracks | Feature tracks | Quantitative tracks | Click/hover on features | Change feature drawing density | Multi-line mode | Open-source | FF support | Safari/Chrome support | IE support | Custom track rendering |
| --- | --- | --- | --- | --- | --- | --- | --- | --- | --- | --- | --- | --- | --- | --- |
| UCSC Genome Browser | d | d | C | r | Y | Y | Y | r | – | Y | Y | Y | Y | s |
| Ensembl Genome Browser | d | d | C | d | Y | Y | Y | d | – | Y | Y | Y | Y | s |
| NCBI Sequence Viewer 2.19 | C | d | d | d | Y | Y | Y | d | – | – | Y | Y | Y | s |
| JGI Genome Browser | r | r | C | r | Y | Y | Y | r | – | – | Y | Y | Y | s |
| GBrowse 2.0 | d* | d | C | C | Y | Y | Y | d | – | Y | Y | Y | Y | s |
| Anno-J | C | d | C | C | Y | Y | Y | C | – | Y | Y | Y | – | – |
| JBrowse | C | C | C | C | Y | Y | Y | – | – | Y | Y | Y | Y | – |
| ABrowse | C | D | C | C | Y | Y | Y | – | – | Y | Y | Y | Y | s |
| ChromoZoom | IC | C | C | C | Y | Y | Y | C | Y | Y | Y | Y | Y^†^ | B |

These comparisons were valid at the time of writing, and may change by the time of publication. Symbols—Y: supported, –: not supported, r: page reloads required, d: no page reload required, but transition is discontinuous, C: no page reload required and transition is continuous, s: server-side, B: browser-side, I: inertial movement supported. Based on Table 1, Skinner et al. *Genome Res.* **19**: 1630-1638. Notes—*: Continuous for an interval, then a discontinuous transition is required. †: Performance is suboptimal in Internet Explorer and certain HTML5 features are not available.
